# Toward Cholera Elimination, Haiti

**DOI:** 10.3201/eid2711.203372

**Published:** 2021-11

**Authors:** Stanislas Rebaudet, Patrick Dély, Jacques Boncy, Jean Hugues Henrys, Renaud Piarroux

**Affiliations:** Hôpital Européen, Marseille, France (S. Rebaudet);; Aix Marseille Univ, Institut National de la Santé et de la Recherche Médicale, Institut de Recherche pour le Développement, Sciences Économiques et Sociales de la Santé et Traitement de L'information Médicale, ISSPAM, Marseille (S. Rebaudet);; Institut Pierre-Louis d’Epidémiologie et de Santé Publique, Sorbonne Université, Institut National de la Santé et de la Recherche Médicale, Paris, France (S. Rebaudet, R. Piarroux);; Direction d'Epidémiologie des Laboratoires et de la Recherche, Ministère de la Santé Publique et de la Population, Delmas, Haiti (P. Dély);; Faculté de Médecine et de Pharmacie, Université d'Etat d'Haïti, Port-au-Prince, Haiti (P. Dély);; Laboratoire National de Santé Publique, Ministère de la Santé Publique et de la Population, Delmas (J. Boncy);; Equipe de recherche sur l’écologie des maladies infectieuses et tropicales (EREMIT), Université Quisqueya, Port-au-Prince (J.H. Henrys);; AP-HP, Hôpital Pitié-Salpêtrière, Paris (R. Piarroux)

**Keywords:** cholera, Haiti, elimination, vaccination, case-area targeted intervention, rapid response team, mobile team, Vibrio cholerae, bacteria

## Abstract

This study describes the apparent discontinuation of cholera transmission in Haiti since February 2019. Because vulnerabilities persist and vaccination remains limited, our findings suggest that case-area targeted interventions conducted by rapid response teams played a key role. We question the presence of environmental reservoirs in Haiti and discuss progress toward elimination.

After cholera was reintroduced into Haiti in 2010 (*1*), the country experienced an epidemic of unparalleled magnitude: the 9,789 recorded casualties represent the largest number for a single epidemic in the past 20 years (https://apps.who.int/gho/data/node.main.174). Unfortunately, vulnerabilities of people in Haiti to fecal–oral diseases such as cholera have barely been reduced over the past decade. The National Plan for the Elimination of Cholera 2013–2022 aimed to improve access to drinking water to >85% of the population, access to sanitation to >90% of the population, and access to healthcare to >80% of the population (*2*). However, these indicators improved very slowly or even deteriorated during 2012–2017 (*3*). The country still faces a deep economic and social crisis and has also endured several natural disasters, such as Hurricane Matthew in October 2016. In addition, the Multi-Partner Trust Fund set in December 2016 by the United Nations to support the response to cholera in Haiti gathered only US $20.8 million during 2016–2020 (https://mptf.undp.org/factsheet/fund/CLH00).

To alleviate these persisting vulnerabilities and eliminate cholera transmission, experts and public health institutions have appealed to expand mass use of oral cholera vaccines (OCV) (*4*,*5*). Meanwhile, a nationwide coordinated rapid response strategy structured around case-area targeted interventions was gradually implemented beginning in July 2013 by the Ministry of Public Health and Population of Haiti (MOH), UNICEF, and other partners (*6*). Analogous to forest fire management, the strategy aimed to rapidly detect local outbreaks and send rapid response teams, mostly composed of nongovernmental organization and MOH staff, to the households and neighbors of infected persons (Appendix). This study aims to describe and decipher the progress of cholera control in Haiti.

## The Study

**Figure 1:**
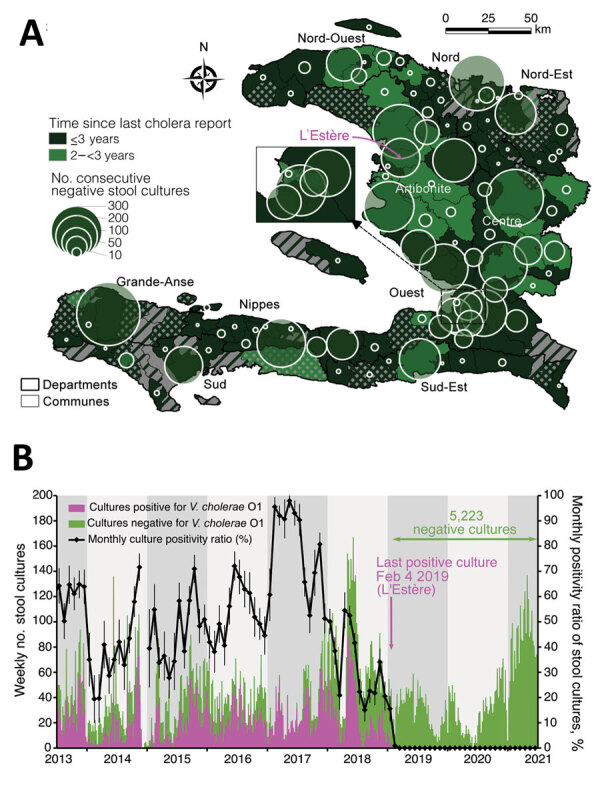
Cholera elimination progress and surveillance effort in Haiti as of July 1, 2021. A) Time elapsed since the last cholera report (i.e., number of years since the last positive culture or last reported suspected cholera case [choropleth colors and patterns]) and of the number of consecutive negative cultures (proportional circles), by commune. Communes with >1 negative culture since the last positive culture or the last reported death are colored with solid green, with elimination time calculated since the last positive culture or suspected cholera death; communes with no stool sampled for culture since the last positive culture or the last reported death are colored with green cross-hatching, with elimination time calculated since the last reported suspected cholera case or death; communes with no history of stool sampling for cholera culture but with reported cases are colored with green diagonal hatching, with elimination time calculated since the last reported suspected cholera case or death; and communes with no history of stool sampling and no reported cases are colored in solid gray. Communes are colored according to the time elapsed since possible elimination (i.e., number of years since the last positive culture or the last reported suspected cholera case). The magenta arrow localizes the commune of the last positive stool sample in Haiti. B) Plot of the weekly number of positive (magenta) and negative (green) stool cultures for *Vibrio cholerae* O1 and monthly culture-positivity ratio. Data source: Ministry of Public Health and Population of Haiti (pers. comm., 2021 Jul 20; see also Appendix). *V. cholerae* O1, *Vibrio cholerae* O1.

We analyzed cholera surveillance data routinely collected since 2010 by the MOH with support of the Pan American Health Organization and the Centers for Disease Control and Prevention, including results of stool cultures searching for *Vibrio cholerae* O1 (Appendix). The study was approved by the Bioethics National Committee of the MOH (authorization no. 1819-41). Suspected cholera cases stagnated during 2013–2016; the median was 38,733 annual cases (incidence rate 6.9/100,000 person-weeks). Incidence then dramatically decreased to 13,681 cases (incidence rate 2.2/100,000 person-weeks) in 2017, to 3,777 cases (incidence rate 0.6/100,000 person-weeks) in 2018, and to 720 cases in 2019 (incidence rate 0.1/100,000 person-weeks). The last cluster of suspected cholera cases and the last cholera-associated death were observed in the commune of L’Estère, Artibonite department, in February 2019. As of July 1, 2021, a total of 92 (67%) of the 140 communes have not notified a case for >3 years (Figure 1, panel A). Of note, the last stool culture positive for *V. cholerae* O1 was also sampled in L’Estère on February 4, 2019; none of the 5,223 consecutive stool specimens sampled from diarrheic patients across the country, including 2,255 specimens sampled in 2021, have tested positive since 2019 (Figure 1, panel B). Reports of cholera have thus halted for >3 years in 112 (80%) of communes in Haiti (Figure 1, panel A).

**Figure 2:**
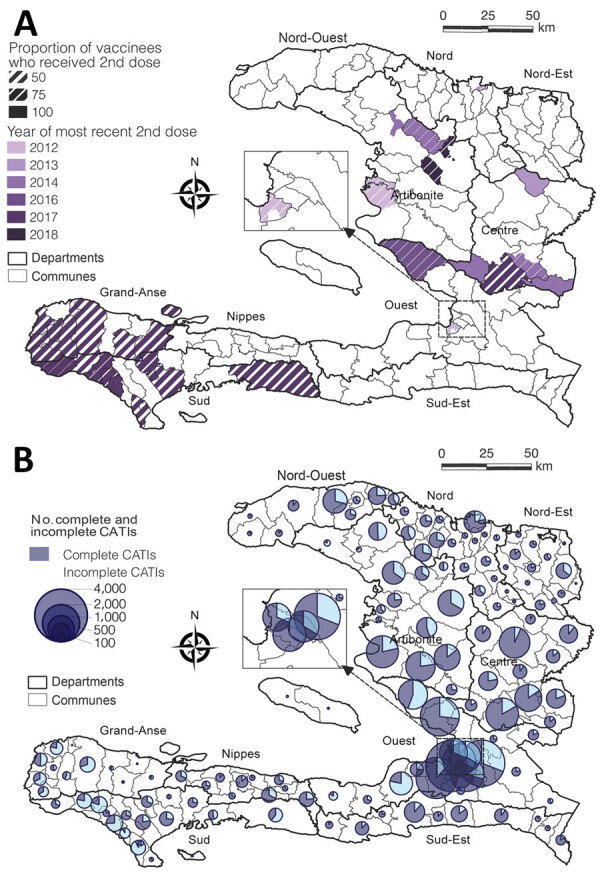
Cholera control in Haiti, 2012–2019. A) Oral cholera vaccine campaigns during 2012–2018 by subcommune; B) complete and incomplete CATIs conducted during July 2013–December 2019 by commune. Complete CATIs are defined by house decontamination, education, soap and chlorine distribution, and distribution of antibiotics to close contacts of cholera case-patients. Data source: Ministry of Public Health and Population of Haiti (pers. comm., 2021 Jul 20); UNICEF (pers. comm., 2020 Jan 20; see also Appendix). CATI, case-area targeted interventions.

To analyze factors associated with this apparent discontinuation of cholera transmission in February 2019, we compiled data from mass OCV campaigns implemented across Haiti (Appendix). During 2012–2018, the MOH recorded 33 campaigns targeting 31 communes and 16 prisons (Table 1; Figure 2, panel A). A total of 1,576,209 persons received >1 dose. The 2-dose regimen was completed for 74% of these persons, with marked heterogeneity (Figure 2, panel A). Overall, <10% of the population in Haiti has been fully vaccinated (Table 1). Considering the duration of protection of 1-dose and 2-dose regimens of OCVs (*7*), only 2.4% of persons in Haiti were likely still protected in 2019 (Table 1).

**Table 1:** Summary of killed whole-cell oral cholera vaccine campaigns, Haiti, 2012–2019*

Year	Population in Haiti	No. (%) targeted communes	No. persons who received >1 OCV dose	No. persons who received 2nd OCV dose (%)	Percentage of fully vaccinated population†	Percentage of population with residual vaccine immunity‡
2012	10,644,927	3	97,774	88,762	0.8%	0.5%
2013	10,937,675	2	113,045	102,250	0.9%	1.0%
2014	11,239,398	8	197,147	188,909	1.7%	1.8%
2015	11,550,392	0	0	0	0.0%	1.5%
2016	11,870,966	18	885,210	106,054	0.9%	1.5%
2017	12,201,437	3	215,358	628,049	5.1%	5.1%
2018	12,542,135	1	67,675	59,537	0.5%	3.9%
2019	12,893,402	0	0	0	0.0%	2.4%
Total	NA	31 (22)	1,576,209	1,173,561 (74)	9.1%	NA

*Data source: Ministry of Public Health and Population of Haiti (pers. comm., 2021 Jul 20; see also Appendix). NA, not applicable; OCV, oral cholera vaccine. †The proportion of fully vaccinated population was calculated by dividing the number of persons who received the 2nd OCV dose by the population of Haiti. ‡Percentage of the population with residual vaccine immunity was estimated taking into account the number of vaccinated persons, the percentage of fully vaccinated persons, and the published protection duration after a 1-dose and 2-dose regimen (Appendix).

We compiled a total of 48,710 case-area targeted interventions recorded by UNICEF during July 2013–December 2019 that were implemented across 139 administrative communes (Table 2; Figure 2, panel B). Of those interventions, ≈71% involved a complete package: house decontamination by chlorine spraying, health education about cholera, distribution of soap and chlorine tablets for household water treatment, and distribution of antibiotic prophylaxis to close contacts of cholera cases. Progress from 2013 to 2019 was strong (Table 2), and spatial heterogeneity was marked (Figure 2, panel B). The overall number of case-area targeted interventions per suspected cholera case was 0.3; this ratio improved markedly during 2013–2019 (Table 2).

**Table 2:** Suspected cholera cases and case-area targeted interventions, Haiti, 2012–2019*

Year	Total no. suspected cholera cases	No. CATIs	No. (%)		CATIs/case ratio‡
			Complete CATIs†	Targeted communes	
2012	101,503	ND	ND	ND	ND
2013	58,574	3,599	4 (0)	87 (62)	0.1
2014	27,392	3,241	434 (13)	125 (89)	0.1
2015	36,045	8,091	5,500 (68)	131 (94)	0.2
2016	41,421	13,031	10,869 (83)	138 (99)	0.3
2017	13,681	12,244	10,739 (88)	129 (92)	0.9
2018	3,777	6,561	5,525 (84)	83 (59)	1.7
2019	458	1,943	1,683 (87)	42 (30)	4.2
Total	181,348	48,710	34,754 (71)	139 (99)	0.3

*Data source: UNICEF (pers. comm., 2020 Jan 20; see also Appendix). Exhaustive recording of CATIs by UNICEF started in July 2013 with the launch of the nationwide coordinated rapid response strategy. CATI, case-area targeted interventions; ND, no data. †CATI with house decontamination, education, soap and chlorine distribution, and distribution of antibiotic drugs to contacts. ‡Ratio of the total number of CATIs by the total number of suspected cholera cases, per year.

## Conclusions

As confirmed by an extensive laboratory-based surveillance effort, despite sociopolitical turmoil, the cholera epidemic in Haiti seems to be ending. However, a high-coverage national 2-dose cholera vaccination campaign could not be implemented, because neither the required stockpile nor the funds, estimated at US $66 million (*5*), have been available. Although OCV campaigns proved effective in some targeted areas (*8*), these limited and incomplete campaigns were insufficient to compensate for the global waning of the herd immunity built up during the initial incidence peaks of 2010–2012. In the absence of major progress in water, sanitation, and hygiene indicators, most of the fight against cholera transmission has thus been conducted through the nationwide rapid response strategy, which was gradually implemented beginning in mid-2013 (*6*) and was shown to effectively shorten and mitigate cholera outbreaks in Haiti (*9*).

According to observational and experimental results from Bangladesh, vibriophages might play a role in the natural control of cholera epidemics (*10*). Although a single phage isolation was reported in Haiti in 2013 (*11*), vibriophages might have influenced the seasonal dynamic of cholera. Whether they have also contributed to the epidemic collapse requires further investigation.

Because reports of long-term carriers of cholera are anecdotal and they have not been shown to trigger outbreaks (*12*), the critical issue now is whether the epidemic strain of *V. cholerae* O1 has settled in Haiti and could lead to the reemergence of cholera in the near future (*13*). This scenario explicitly informed the Elimination Plan, which required substantial progress in human development to limit the annual incidence rate of cholera to 0.01% of the population (*2*). According to the published literature (*13*,*14*), no epidemic strain seems to have been isolated in surface waters in Haiti since November 2015. Until then, environmental isolates had remained sporadic and usually concomitant to local cholera cases; therefore, differentiating a true environmental reservoir from a recent fecal contamination remains controversial (*13*,*14*). Cholera recurrence after lull periods might simply come from a low-grade and underreported persistent interhuman transmission (*14*) rather than from aquatic reservoirs (*13*).

Because cholera has not been reported in the neighboring Dominican Republic since 2018 (http://digepisalud.gob.do/documentos), the island of Hispaniola might now be located thousands of kilometers away from current transmission foci. In the past, numerous countries in Africa have experienced severe epidemics and prolonged remissions, despite low human development indices, and have remained free from cholera for years (e.g., 8 years for Guinea and 19 years for Madagascar). Similarly, the absence of cholera outbreaks in South America since the early 2000s, despite a large epidemic wave in the 1990s, is reason for optimism.

Until the certification of cholera elimination by the World Health Organization, systematic bacteriologic testing of every case of severe acute watery diarrhea, combined with environmental monitoring, should be maintained in Haiti. However, achieving 2.5 years with no deaths from cholera or confirmed cholera cases in a country where the disease was considered impregnable is already a victory. This achievement should be considered a springboard to further understand cholera epidemics and improve control strategies worldwide. This success should foster investments in water, sanitation, and hygiene infrastructure, which will protect Haiti against possible future cholera epidemics and against other remaining waterborne diseases.

Appendix Additional information on cholera elimination in Haiti.
