# Supplementary material for: Toward Cholera Elimination, Haiti
Source: Emerg Infect Dis. 2021 Nov;27(11):2932–6. doi: 10.3201/eid2711.203372 (PMC8544981; doi:10.3201/eid2711.203372)
Supplement: Appendix — Additional information on cholera elimination in Haiti. [file 20-3372-Techapp-s1.pdf]

Article DOI: https://doi.org/10.3201/eid2711.203372

Toward Cholera Elimination, Haiti

Appendix

Description of Cholera Surveillance System in Haiti

Since October 2010, cholera treatment facilities (between 136 and 262 cholera treatment centers, cholera treatment units, and acute diarrhea treatment centers, depending on the period) routinely recorded and notified cholera-associated illnesses and deaths. According to the World Health Organization standard definition (*1*), a probable cholera case was defined as acute watery diarrhea with or without vomiting in a patient ≥5 years of age. In Haiti, suspected cholera cases in children <5 years old with similar symptoms were also separately recorded and included in the global cholera toll. Daily suspected cases (both hospitalized and nonhospitalized), as well as daily suspected cholera deaths in health facilities and the community, were anonymously transmitted to 1 of the 10 department health directorates through formatted text message (*2*) or telephone call. Department directorates compiled and validated this data by using Microsoft Excel (https://www.microsoft.com), then sent the data to the national Directorate of Epidemiology Laboratory and Research, typically daily, albeit with frequent delays. The Directorate of Epidemiology Laboratory and Research validated departmental data and compiled the information into a national spreadsheet at a daily and communal scale before performing analyses. Commune records noted the treatment facility of cholera patients, not their place of residence. Community cases were not recorded. By January 2020, a total of 820,497 suspected cholera cases and 9,582 associated deaths had been recorded by the Ministry of Public Health and Population of Haiti (MOH).

Since 2010, routine bacteriologic confirmation of suspected cholera cases was performed at the National Laboratory of Public Health (LNSP) in Delmas, Haiti, by using standard sampling, culture, and phenotyping methods (*3*); the Centers for Disease Control and Prevention provided support. Since July 2017, a private laboratory run by the nongovernmental organization (NGO) Zanmi Lasante and located in Saint-Marc, Artibonite department, performed cultures of stool specimens with support of UNICEF and notified results to the LNSP. During October

2010–June 2021, a total of 24,610 stool specimens, mostly sampled using rectal swab specimens in Carry-Blair transport medium, have been included in the national microbiologic cholera surveillance system. Overall, 40.6% of the specimens were positive for *Vibrio cholerae* O1.

Brief Description of Cholera Control Interventions in Haiti

From 2012 onward, the MOH and its partners have organized several oral cholera vaccine (OCV) campaigns using 2-dose killed whole-cell OCVs: Shanchol (Shantha Biotechnics, https://www.sanofi.in), then Euvichol (Eubiologics, http://eubiologics.com). In 2012, 2 pilot campaigns were implemented in an urban neighborhood of the capital Port-au-Prince (*4*) and in a rural area of the Artibonite department (*5*). In the years after, several additional campaigns were conducted across the country, notably in Cap Haitien, the second largest city in the country, in 2013, and several highly affected communes of the central area (*6*,*7*). For fear of a cholera upsurge after Hurricane Matthew, which hit the southwestern tip of Haiti in October 2016, a large OCV campaign targeting >800,000 residents was launched in November 2016 in 18 communes of the Sud and Grande-Anse departments. However, the second dose was administrated only in May–June 2017. The last OCV campaign implemented in Haiti targeted several areas of Saint-Michel-de-l'Attalaye, a highly affected commune in the Artibonite department, in April–May 2018.

Beginning in July 2013, the nationwide case-area targeted rapid response strategy to eliminate cholera in Haiti was laboriously but increasingly implemented throughout the country (*8*). In all departments, UNICEF established partnerships with NGOs that hired water, sanitation, and hygiene rapid response teams composed of staff local to Haiti. The MOH also established its own teams called EMIRAs (Equipe mobile d'intervention rapide), which included healthcare workers (i.e., nurses and auxiliary nurses). Staff of the NGO rapid response teams and EMIRA worked together and deployed mixed teams, which were requested to respond to every suspected cholera case or death within 48 hours of admission to a healthcare facility. For this purpose, rapid response teams were encouraged to obtain lists of cholera cases on a daily basis from department health directorates and treatment centers (*8*). The core methodology of case-area targeted interventions (CATIs) had been established with the MOH and its partners and has been described elsewhere (*8*). In brief, it included door-to-door visits to affected families and their

neighbors; house decontamination by chlorine spraying of latrines and other potentially contaminated surfaces; onsite organization of education sessions about cholera and hygiene promotion; distribution of 1 cholera kit per household (composed of soaps, sachets of oral rehydration solution, and chlorine tablets; prophylactic antibiotics with doxycycline to contacts living with cholera case-patients; manual bucket chlorination at drinking water collection points, when appropriate; chlorinated water supply systems; supervision of safe funeral practices for cholera deaths; and primary care to cholera case-patients in the community. CATIs were prospectively documented and transmitted by water, sanitation, and hygiene rapid response teams to UNICEF with date, location (i.e., commune, communal section, and locality) and implemented activities, including specific activities of embedded EMIRA staff.

Data Collection and Analysis

We first used anonymous information of daily reported numbers of suspected cholera cases and deaths at the communal level (Ministry of Public Health and Population of Haiti, pers. comm., 2021 Jul 20); the study was authorized by the Bioethics National Committee of the MOH (authorization no. 1819–41). Annual population estimates were extrapolated from the 2012 and 2015 estimates provided by the Haitian Institute of Statistics and Informatics (*9*,*10*). We then computed annual cholera incidence rates. We also manually searched the database for most recent clusters of suspected cholera cases and compared that data with prospective weekly cholera epidemiologic bulletins of the MOH (https://mspp.gouv.ht/newsite/_direction/pageDocumentation.php?IDDir = 9, cited 2020 Dec 14). For each commune, we computed the time elapsed since the last recorded suspected cholera case.

As prevalence of cholera decreases, the predictive positive value of the clinical definition for suspected cholera cases weakens (*11*), and stool culture surveillance for *Vibrio cholerae* O1 becomes more vital to follow the dynamic of the epidemic. We thus analyzed the LNSP database of cholera stool cultures (Ministry of Public Health and Population of Haiti, pers. comm., 2021 Jul 20) and plotted the weekly number of positive and negative cultures, as well as the monthly culture-positivity rate. For each of the administrative communes of Haiti, we also calculated the time since the last positive stool culture, time since the last negative culture, and the number of

consecutive negative cultures since the last positive one. We also calculated the time since the last suspected cholera death and the last suspected cholera case. We then classified the surveillance effort for each commune and calculated the time since possible cholera elimination accordingly (Appendix Table). Finally, we counted the number of communes with possible cholera elimination for ≥3 years, which corresponds to the definition of cholera elimination according to the Global Task Force on Cholera Control (*12*), and mapped these indicators (Appendix Table).

To analyze factors associated with the cholera dynamic, we summarized OCV campaigns recorded by the MOH, including date, place at the subcommunal level (called section communale in Haiti), number of persons who received ≥1 dose, and number of persons who received 2 doses. We mapped these OCV campaigns at the subcommunal level, illustrating the administration year of the 2nd dose and the proportion of vaccinees who received the 2nd dose. By using Haitian Institute of Statistics and Informatics demographic estimates, we approximated the proportion of the population in Haiti who were fully vaccinated since 2012. Because protection of OCVs decreases over time (*13*), we approximated the annual proportion of the population with residual vaccine immunity by multiplying the number of persons vaccinated during each campaign by the published vaccine effectiveness for a 2-dose regimen (56% the first year, 59% the second year, 39% the third year, and 24% the fourth year) and 1-dose regimen (40% the first year) (*13*). We considered effectiveness dropped to 0% after these delays.

Finally, we summarized CATIs recorded by UNICEF between the onset of the nationwide strategy in July 2013 and December 2019 (UNICEF, pers. comm., 2020 January 20). We counted the number of CATIs by commune and by year, as well as the number and proportion of CATIs that involved a complete package of house decontamination by chlorine spraying, health education about cholera, distribution of soap and chlorine tablets for household water treatment, and distribution of antibiotic prophylaxis to close contacts of cholera case-patients. We then mapped the number of CATIs by commune with the proportion of complete CATIs. To assess the evolution of the relative response effort to suspected cholera cases, we calculated the annual ratio of the total number of CATIs to the total number of suspected cholera cases.

Data management was performed using Microsoft Excel for Mac version 16.45. QGIS version 3.8 (http://www.qgis.org) was used to draw the maps.

References

1. Global Task Force on Cholera Control. Cholera outbreak: assessing the outbreak response and improving preparedness. Geneva, Switzerland: World Health Organization; 2004 [cited 2021 Sep 29]. http://www.who.int/publications/i/item/cholera-outbreak-assessing-the-outbreak-response-and-improving-preparedness

2. Pan American Health Organization/World Health Organization. New mobile phone-based system facilitates cholera surveillance and control in Haiti. 2013 Feb 26 [cited 2021 Sep 29]. https://www3.paho.org/ict4health/index.php?option=com_content&view=article&id=169:nuevo-sistema-basado-en-telefonia-movil-facilita-la-vigilancia-y-el-control-del-colera-en-haiti&Itemid=204&lang=en

3. Centers for Disease Control and Prevention. Laboratory methods for the diagnosis of epidemic dysentery and cholera. Atlanta, Georgia: Centers for Disease Control and Prevention; 1999 [cited 2021 Sep 29]. http://www.cdc.gov/cholera/pdf/Laboratory-Methods-for-the-Diagnosis-of-Epidemic-Dysentery-and-Cholera.pdf

4. Rouzier V, Severe K, Juste MAJ, Peck M, Perodin C, Severe P, et al. Cholera vaccination in urban Haiti. Am J Trop Med Hyg. 2013;89:671–81. PubMed https://doi.org/10.4269/ajtmh.13-0171

5. Ivers LC, Teng JE, Lascher J, Raymond M, Weigel J, Victor N, et al. Use of oral cholera vaccine in Haiti: a rural demonstration project. Am J Trop Med Hyg. 2013;89:617–24. PubMed https://doi.org/10.4269/ajtmh.13-0183

6. Poncelet JL. Cholera in Haiti: successes and challenges in a low income country. Wkly Epidemiol Rec. 2015;90:542–4. PubMed

7. Ivers LC. Eliminating cholera transmission in Haiti. N Engl J Med. 2017;376:101–3. PubMed https://doi.org/10.1056/NEJMp1614104

8. Rebaudet S, Bulit G, Gaudart J, Michel E, Gazin P, Evers C, et al. The case-area targeted rapid response strategy to control cholera in Haiti: a four-year implementation study. PLoS Negl Trop Dis. 2019;13:e0007263. PubMed https://doi.org/10.1371/journal.pntd.0007263

9. Institut Haitien de Statistique et d'Informatique (IHSI), Direction des Statistiques Démographiques et Sociales, Ministère de l'Economie et des Finances, République d'Haïti. Total population, population of 18 years and older, households and densities estimated in 2012 [in French]. Port-au-Prince, Haiti; 2012 [cited 2021 Sep 29]. https://www.humanitarianresponse.info/sites/www.humanitarianresponse.info/files/documents/files/Estimation%20et%20projection%20de%20la%20population%202012.pdf

10. Institut Haitien de Statistique et d'Informatique (IHSI), Direction des Statistiques Démographiques et Sociales, Ministère de l'Economie et des Finances, République d'Haïti. Total population, population of 18 years and older, households and densities estimated in 2015 [in French]. Port-au-Prince, Haiti; 2015 [cited 2021 Sep 29]. https://www.humanitarianresponse.info/sites/www.humanitarianresponse.info/files/documents/files/estimat_poptotal_18ans_menag2015.pdf

11. Brenner H, Gefeller O. Variation of sensitivity, specificity, likelihood ratios and predictive values with disease prevalence. Stat Med. 1997;16:981–91. PubMed https://doi.org/10.1002/(SICI)1097-0258(19970515)16:9<981::AID-SIM510>3.0.CO;2-N

12. Global Task Force on Cholera Control. Ending cholera: a global roadmap to 2030. World Health Organization; 2017 [cited 2021 Sep 29]. https://www.who.int/cholera/publications/global-roadmap.pdf

13. Bi Q, Ferreras E, Pezzoli L, Legros D, Ivers LC, Date K, et al.; Oral Cholera Vaccine Working Group of The Global Task Force on Cholera Control. Protection against cholera from killed whole-cell oral cholera vaccines: a systematic review and meta-analysis. Lancet Infect Dis. 2017;17:1080–8. PubMed https://doi.org/10.1016/S1473-3099(17)30359-6

Appendix Table. Category of cholera surveillance effort and associated definition of the time since possible cholera elimination, by commune.

Category of cholera surveillance effort		Definition of the time since possible cholera elimination
(a)	Communes with ≥1 negative culture since the last positive culture or the last reported death	Time elapsed since the last positive culture or suspected cholera death
(b)	Communes with no culture since the last positive one or the last reported death	Time since the last reported suspected cholera case or death
(c)	Communes with no history of stool sampling for cholera culture but with reported cases	Time since the last reported suspected cholera case or death
(d)	Communes with no history of stool sampling and no reported cases	No definition
